# Epidemiological characterization of SARS-CoV-2 variants in children over the four COVID-19 waves and correlation with clinical presentation

**DOI:** 10.1038/s41598-022-14426-0

**Published:** 2022-06-17

**Authors:** Claudia Alteri, Rossana Scutari, Valentino Costabile, Luna Colagrossi, Katia Yu La Rosa, Emanuele Agolini, Valentina Lanari, Sara Chiurchiù, Lorenza Romani, Anna Hermine Markowich, Paola Bernaschi, Cristina Russo, Antonio Novelli, Stefania Bernardi, Andrea Campana, Alberto Villani, Carlo Federico Perno

**Affiliations:** 1 Multimodal Research Area, Bambino Gesù Children Hospital IRCCS, Rome, Italy; 2 Department of Oncology and Hemato-Oncology, University of Milan, Milan, Italy; 3 Microbiology and Diagnostics of Immunology Unit, Bambino Gesù Children Hospital IRCCS, Rome, Italy; 4 Academic Department of Pediatrics, Bambino Gesù Children’s Hospital, IRCCS, Rome, Italy; 5 Laboratory of Medical Genetics, Translational Cytogenomics Research Unit, Bambino Gesù Children Hospital IRCCS, Rome, Italy

**Keywords:** SARS-CoV-2, Viral genetics, Clinical microbiology

## Abstract

Since the start of SARS-CoV-2 pandemic, children aged ≤ 12 years have always been defined as underrepresented in terms of SARS-CoV-2 infections’ frequency and severity. By correlating SARS-CoV-2 transmission dynamics with clinical and virological features in 612 SARS-CoV-2 positive patients aged ≤ 12 years, we demonstrated a sizeable circulation of different SARS-CoV-2 lineages over the four pandemic waves in paediatric population, sustained by local transmission chains. Age < 5 years, highest viral load, gamma and delta clades positively influence this local transmission. No correlations between COVID-19 manifestations and lineages or transmission chains are seen, except for a negative correlation between B.1.1.7 and hospitalization.

## Introduction

Since the start of the 2019 coronavirus disease pandemic (COVID-19), it was clear the importance of a sustained monitoring and rapid assessment of SARS-CoV-2 transmission dynamics, to be rapidly aware of increased transmissibility, of increased virulence or change in clinical disease presentation, and of decrease in effectiveness of public health and social measures of a specific variant^1^. All studies aimed to define these aspects were based on a SARS-CoV-2 infected population, at the beginning of the pandemic mostly composed (due to availability of tests only for clinically advanced people) of adult and older age groups, being at higher risk of serious COVID-19 manifestations^2,3^.

As of the beginning of December 2021, no studies have characterised the circulation of SARS-CoV-2 variants in newborns and children over the four pandemic waves. Consequently, there is not enough information about the SARS-CoV-2 transmission trajectories in children, how different SARS-CoV-2 variants may affect different ages, and/or may change transmission or patterns of diseases.

Children and adolescents usually experience less severe COVID-19 manifestations^4,5^. Most children exposed to SARS-CoV-2 remain asymptomatic or develop mild, upper respiratory tract symptoms which resolve within a few days^6,7^. Reports of hospitalization and severe long-term manifestations are less frequent than adults, but not negligible. Severe and multiple COVID-19 related illnesses were indeed reported in young people who test positive for SARS-CoV-2 by several independent groups^6–11^. Multisystem inflammatory syndrome (MIS-C), and myocarditis were the long-term COVID-19 related manifestations, mostly related with young age^8–11^.

Children were also underrepresented in term of global SARS-CoV-2 prevalence respect to adult population^12^. Indeed, according with age, the global SARS-CoV-2 in children encompasses the 1.8% in under-5 years population to 6.3% of population aged from 5 to 14 years^12^. More recently, an increased transmissibility across all age groups has been reported for SARS-CoV-2 variants of concerns (VOCs), most notably for the Delta variant^13,14^. However, due to the modest COVID-19 related symptoms, it cannot be excluded that in the first months of the pandemic, when the diagnosis was mainly symptom-based, a substantial part of SARS-CoV-2 positive children escaped the SARS-CoV-2 screening strategies^15^, thus causing an underestimation of SARS-CoV-2 in this population.

Here, our objectives are to fill gaps in the knowledge of SARS-CoV-2 paediatric epidemic thanks to the viral definition, transmission dynamics and clinical characterization of more than 600 children diagnosed with SARS-CoV-2 infection in a large, scientific paediatric institute for research, hospitalization and healthcare in the Central Italy. With an integrated approach comprising epidemiological, clinical and viral genetic data, this study provides a unique data set to better understand temporal evolutions of SARS-CoV-2 in children and to identify any changes in clinical manifestations from emerged SARS-CoV-2 variants.

## Methods

### Sample collection and study design

This retrospective observational study originally included 731 SARS-CoV-2-positive nasopharyngeal-swabs, obtained from COVID-19 patients aged ≤ 12 years referred to Bambino Gesù Children Hospital IRCCS (OPBG) during the period of March 05, 2020, to August 31, 2021. The SARS-CoV-2-positive nasopharyngeal-swabs were selected according to the criteria defined in Supplementary Fig. 1. Only 612 samples were finally selected cause successfully sequenced.

Demographics, epidemiological and clinical data were obtained retrospectively by pseudonymized electronic medical records. The study protocol was approved by local Research Ethics Committee of OPBG (prot. 2384_OPBG_2021). This study was conducted in accordance with the principles of the 1964 Declaration of Helsinki. Informed consent was waived in accordance with the hospital regulations on observational retrospective studies.

Severity of SARS-CoV-2 infection was defined according to Dong Y, et al., Pediatrics. 2020^16^, and based on the clinical features, laboratory testing, and chest radiograph imaging in our cohort. The following definitions were used: (i) asymptomatic infection defined as children tested SARS-CoV-2 positive after a contact tracing but not developing any clinical symptoms; (ii) mild infection defined as symptoms of upper respiratory tract infection, such as cough, sore throat, runny nose, and sneezing, that may include also fever, fatigue, myalgia, and/or symptoms of gastrointestinal tract infection, defined as vomiting, abdominal pain, nausea or diarrhoea; (iii) moderate/severe infection defined as symptoms of lower respiratory tract infection including clinical signs of bronchitis or pneumonia (fever, cough, dyspnoea, fast breathing) with or without signs of gastrointestinal symptoms. Patients developing dyspnoea and with oxygen saturation < 92% were included in this category.

At the time of diagnosis, SARS-CoV-2 RNA was quantified by a ddPCR home-made protocol targeting three different RNA polymerase RNA dependent (RdRp) regions^17^. Quantitative results were then normalized in number of copies per mL.

### Virus amplification and sequencing

Viral RNAs were extracted from nasopharyngeal swabs by using QIAamp Viral RNA Mini Kit, followed by purification with Agencourt RNAClean XP beads. Both the concentration and the quality of all isolated RNA samples were measured and checked with the Nanodrop. Amplicons of whole genome sequences of SARS-CoV-2 were generated with a 50 ng viral RNA template, by using a multiplex approach e.g. CleanPlex SARS-CoV-2 Research and Surveillance Panel, and QIAseq DIRECT SARS-CoV-2 Kit^18,19^ according to the manufacturer’s protocol. Libraries were then generated using the Nextera DNA Flex library preparation kit with Illumina index adaptors and sequenced on a MiSeq instrument (Illumina, San Diego, CA, USA) with 2 × 150-bp paired-end reads.

Reference-based assembly of the raw data was performed according to^20^. 612 consensus sequences were generated using the GitHub freely distributed software vcf_consensus_builder^21^. All SNPs having a minimum supporting read frequency of 40% with a depth ≥ 10 were retained.

### Phylogenetic analysis

SARS-CoV-2 lineages of the 612 SARS-CoV-2 consensus sequences obtained were assigned according to the PANGOLIN application (Pangolin https://pangolin.cog-uk.io/)^22^. In order to describe the relatedness of the paediatric sequences against SARS-CoV-2 diversity, 1233 sequences from GISAID (GISAID sequences) were selected against location and sampling date, and 410 SARS-CoV-2 sequences (local sequences) belonged to adolescent and adult population (> 12 years) living in the same area of the study paediatric population were added.

Sequences were aligned using ClustalX and manually checked using Bioedit. The final alignment had 2255 sequences of 28,655 nucleotides long. Alignment positions showing significant homoplasy were identified by combined approaches in order to account for regions that might potentially be the result of hypervariability or sequencing artifacts. Homoplasies were firstly identified using HomoplasyFinder, and then confirmed by Treetime (homoplasy setting)^23,24^.

In order to explore the phylogenetic structure of the epidemic affecting population aged ≤ 12, a maximum likelihood (ML)^25–27^ phylogeny tree was firstly performed by using the full alignment composed of GISAID, local population, and paediatric sequences. The ML phylogeny tree was built with IqTree^25^ using the best-fit model of nucleotide substitution GTR + I + γ^26^. Tree topology was assessed with the fast-bootstrapping function with 1000 replicates. The ML tree was inspected in TempEst,^27^ in order to define the correlation between genetic diversity (root-to-tip divergence) and time of sample collection (Supplementary Fig. 2). Local clusters of SARS-CoV-2 sequences were defined by an intra-genetic distance < 0.0002 and a bootstrap support ≥ 99.0%.

Bayesian coalescent methods^28,29^ were further performed, in order to define the phylogenetic structure of the paediatric epidemic against time, and to confirm potential transmission chains and clusters. A first Bayesian coalescent tree analysis was undertaken with BEAST v1.10.5^28^, using the GTR + I + γ^26^ substitution model with an exponential population growth tree prior and strict molecular clock, under a noninformative continuous-time Markov chain (CTMC) reference prior^29^ using only paediatric sequences. The most informative sequences for virus spread and clustering identified in the first Bayesian tree and in the ML tree were incorporated in a second Bayesian tree interference, in order to yield more robust reconstructions of transmission clusters (defined by a posterior probability support = 1). Four independent chains were run for 50 million states and parameters and trees were sampled every 1,000 states. Upon completion, chains were combined using LogCombiner after removing 10% of states as burn-in and convergence was assessed with Tracer (ESS > 100). Taxon sets were defined and used to estimate the posterior probability of monophyly and the posterior distribution of the tMRCA of observed phylogenetic clusters.

iTOL^30^ was used to annotate phylogenetic trees with information regarding lineages, clusters, symptoms, hospitalization, and SARS-CoV-2 viral load.

### Statistical analysis

Likelihood Ratio Test, followed by a multinomial logistic regression model to estimate 95% confidence intervals of odds ratios, was used to compare demographic and clinical findings between general and selected SARS-CoV-2 infected populations.

Kruskal–Wallis and Chi-squared test for trend were used to estimate significant changes among different lineages and transmission clusters. Mann–Whitney test and Fisher exact test were used to estimate significant changes between in and out cluster sequences.

A multivariable logistic regression analysis was performed to evaluate demographic, virus-related, and clinical factors independently associated with clustering and hospitalization.

Two-sided *p*-values were always reported.

Data were analyzed using Rgui and the statistical software package SPSS (v32.0; SPSS Inc., Chicago, IL).

## Results

### Patients’ characteristics

From March 05, 2020, through August 31, 2021, nasopharyngeal swabs taken from a total of 45,573 individuals aged ≤ 12 years were screened for SARS-CoV-2 infection at the main paediatric Hospital in Rome (Supplementary Fig. 1). A COVID-19 diagnosis was made for 2399 of them, with a positivity rate that was 0.6% between March and July, 2020, 5.2% between August and December 2020, 5.9% between January and May 2021, and 3.2% between June and August 2021. Whole genome sequencing was performed in 731 samples collected from 731 individuals with varying disease symptoms involving upper or lower respiratory or gastrointestinal tracts. Sampling selection criteria for these samples and the comparison of their demographic and clinical characteristics with SARS-CoV-2 infected ≤ 12 years aged population are illustrated in Supplementary Fig. 1 and 3, Supplementary Table 1 and Supplementary Results. One-hundred and nine samples were excluded due to failed amplification (*n* = 25) or poor genomic coverage (< 60%, *n* = 94). The final study population thus consisted of 612 patients, whose whole genome SARS-CoV-2 were successfully sequenced with their demographic and clinical characteristics reported in Table 1.

**Table 1:** Demographic and clinical characteristics of the 612 SARS-CoV-2-infected patients against lineages.

	Overall *N* = 612	Lineages						*P*-value^§^
		B/B.1/B.1.1	B.1.177 (EU)	B.1.1.7 (Alfa)	P.1/P.1.1 (Gamma)	B.1.617.2/ AY (Delta)	Other^a^	
		*N* = 30 (4.9%)	*N* = 253 (41.3%)	*N* = 127 (20.8%)	*N* = 35 (5.7%)	*N* = 139 (22.7%)	*N* = 28 (4.6%)	
**Demographic characteristics**								
Age, years:	2 (1–6)	5 (1–8)	2 (1–6)	3 (1–6)	1 (1–6)	2 (1–7)	1 (1–7)	0.163
< 1	215 (35.1)	7 (23.3)	100 (39.5)	39 (30.7)	14 (40.0)	43 (30.9)	12 (42.9)	0.228
1–5	217 (35.5)	9 (30.0)	85 (33.6)	54 (42.5)	12 (34.3)	50 (36.0)	7 (25.0)	0.384
≥ 5	180 (29.4)	14 (46.7)	68 (26.9)	34 (26.8)	9 (25.7)	46 (33.1)	9 (32.1)	0.726
Sex, Male	345 (56.4)	16 (53.3)	139 (54.9)	77 (60.6)	21 (60.0)	78 (56.1)	13 (46.4)	0.766
**Origin**								
Caucasian	527 (86.1)	19 (63.3)	222 (87.7)	112 (88.2)	30 (85.7)	122 (87.8)	22 (78.6)	0.631
Asian	30 (4.9)	3 (10.0)	8 (3.2)	5 (3.9)	3 (8.6)	6 (4.3)	5 (17.9)	0.074
North American	10 (1.6)	2 (6.7)	4 (4.6)	1 (0.8)	0 (0.0)	3 (2.2)	0 (0.0)	0.258
Latin American	20 (3.3)	3 (10.0)	5 (2.0)	6 (4.7)	0 (0.0)	6 (4.3)	0 (0.0)	0.056
African	25 (4.1)	3 (10.0)	14 (5.5)	3 (2.4)	2 (5.7)	2 (1.4)	1 (3.6)	0.728
**Residency**								
Lazio	587 (95.9)	26 (86.7)	246 (97.2)	123 (96.9)	32 (91.4)	137 (98.6)	23 (82.1)	0.13
Others^b^/Unknown	25 (4.1)	4 (13.3)	7 (2.8)	4 (3.1)	3 (8.6)	2 (1.4)	5 (17.9)	0.13
Comorbidity^c^	51 (8.3)	5 (16.7)	21 (8.3)	7 (5.5)	2 (5.7)	15 (10.8)	1 (3.6)	0.916
First diagnosis	02.21 (11.20–21.21)	07.20 (03.20–10.20)	11.20 (10.20–01.21)	03.21 (03.21–04.21)	04.21 (03.21–05.21)	08.21 (07.21–08.21)	01.21 (01.21–03.21)	< 0.0001
**Clinical characteristics**								
**COVID-19 at SARS-CoV-2 testing**^**d**^								
Mild^e^	436 (83.2)	17 (58.6)	180 (81.4)	93 (93.9)	25 (83.3)	107 (84.3)	14 (77.8)	0.864
Moderate/severe^f^	51 (9.7)	6 (20.7)	20 (9.0)	4 (4.0)	2 (6.7)	17 (13.4)	2 (11.1)	0.995
Asymtomatic	37 (7.1)	6 (20.7)	21 (9.5)	2 (2.0)	3 (10.0)	3 (2.4)	2 (11.1)	0.797
Hospitalization^g^	107 (19.9)	12 (41.4)	43 (18.9)	8 (7.8)	6 (18.8)	34 (26.6)	4 (22.2)	0.571
Lenght of hospitalization, days^h^	3 (3–10)	7 (4–9)	5 (3–10)	4 (3–12)	6 (4–7)	3 (2–10)	4 (2–8)	0.431
**SARS-CoV-2 rtPCR**^**i**^								
Mean cycle thresholds	18 (15–22)	20 (15–24)	19 (16–23)	18 (15–22)	17 (13–22)	17 (14–20)	20 (16–24)	< 0.0001
E	18 (14–22)	19 (14–24)	19 (16–23)	17 (15–22)	17 (12–21)	15 (13–18)	20 (16–25)	< 0.0001
RdRp	19 (16–23)	20 (15–26)	19 (16–23)	19 (15–22)	17 (12–22)	18 (16–20)	20 (16–25)	0.043
N/N2	18 (15–22)	21 (18–29)	19 (15–22)	18 (15–22)	17 (12–21)	16 (14–19)	20 (17–24)	< 0.0001
SARS-CoV-2 RNA ddPCR log copies/mL	7.7 (6.1–8.5)	6.8 (5.8–8.5)	7.2 (6.1–8.4)	7.7 (6.2–8.5)	8.0 (6.1–8.6)	8.4 (2.3–9.8)	6.6 (5.9–8.1)	< 0.0001
< 6 log copies/mL	106 (17.3)	9 (30.0)	48 (19.0)	24 (18.9)	3 (8.6)	12 (8.6)	10 (35.7)	0.134
> 6–7 log copies/mL	124 (20.3)	6 (20.0)	63 (24.9)	23 (18.1)	9 (25.7)	17 (12.2)	6 (21.4)	0.883
7–8 log copies/mL	148 (24.2)	5 (16.7)	66 (26.1)	34 (26.8)	9 (25.7)	29 (20.9)	5 (17.9)	0.711
8–8.5 log copies/mL	100 (16.3)	4 (13.3)	32 (12.6)	18 (14.2)	4 (11.4)	36 (25.9)	6 (21.4)	0.336
> 8.5 log copies/mL	134 (21.9)	6 (20.0)	44 (17.4)	28 (22.0)	10 (28.6)	45 (32.4)	1 (3.6)	0.452

Data are expressed as median (IQR), or N (%). ^§^Two-sided P-values were calculated by Kruskal–Wallis test, or Chi-square test for trend, as appropriate. ^a^Includes: B.1.160 (*n* = 14), B.1.221 (*n* = 2); B.1.258 (*n* = 4), B.1.351 (*n* = 1), B.1.36 (*n* = 2), B.1.525 (*n* = 2), B.1.398 (*n* = 1), B.1.416 (*n* = 1), C.36.3 (*n* = 1). ^b^Other includes Abruzzo (*n* = 3), Emilia-Romagna (*n* = 3), Lombardia (*n* = 1), Puglia (*n* = 2), Umbria (*n* = 1), Unknown (*N* = 16). ^c^Including respiratory disorders (*n* = 15), cognitive disorders (*n* = 12), oncohematological diseases (*n* = 9), heart diseases (*n* = 7), kidney diseases (*n* = 3), metabolic disorders (*n* = 3), hematological disorders (*n* = 2). ^d^Data available for 524 patients. ^e^Including: symptoms of upper respiratory airways (rinhitis, pharyngo-adenitis, laryngitis) and/or gastrointestinal symptoms. ^f^Including: symptoms of lower respiratory airways (pneumonia, bronchitis and bronchiolitis) with or without gastrointestinal symptoms. ^g^Hospitalization related to COVID-19; data available for 536 patients. ^h^Data available for 122 patients. ^i^Real-time reverse transcription PCR Ct (cycle threshold) values were obtained by AllplexTM 2019-nCoV Assay Seegene (target E, RdRp, N), and Xpert Xpress SARS-CoV-2 Assay, Cepheid (Target E and N2).

Most patients lived in Lazio region (*n* = 587, 95.9%), and were Caucasian (*n* = 527, 86.1%). Three hundred and forty-five (56.4%) were male. The median age was 2 (interquartile range [IQR]: 1–6) years. Two hundred and fifteen (35.1%) patients were under-one year of age. At the time of testing, mild infections were the most prevalent (436 cases, 82.3%), followed by moderate/severe infections (51, 9.7%). Only the 7.1% of patients, identified as a contact of a household case, was asymptomatic. One-hundred and seven patients required hospitalization (19.9%). Four patients, 2 of them aged < 1 year, manifested a severe disease^16^ and required oxygen ventilation in the critical care unit. No deaths were reported.

Symptoms related to upper respiratory airways were most represented (*n* = 445, 85.1%), followed by gastrointestinal symptoms (*n* = 71, 13.5%) and lower respiratory tract symptoms (i.e. bronchitis or pneumonia, *n* = 51, 9.7%). Symptoms did not change substantially among patients with different age ranges, with exception for gastrointestinal symptoms less frequently reported in ≥ 5-year-old children respect to < 1-year-old and 1–5-year-old children (11 [7.7%] vs 36 [18.0%] and 24 [13.3%], *P* = 0.006).

Median (IQR) SARS-CoV-2 nasopharyngeal load was 7.7 (6.1–8.5) log copies/mL. The 62.4% of samples had a SARS-CoV-2 viral load > 7.0 log copies/mL. Viral load was slightly (but significantly) higher in patients aged ≤ 1 year compared to viral load in patients aged 1–5 and ≥ 5 years (8.3 [6.3–8.6] vs. 7.7 [6.2–8.4] vs. 7.1 [6.0–8.3] SARS-CoV-2 log copies/mL, *P* < 0.0001).

By considering the timing of diagnosis, the 53.4% of paediatric SARS-CoV-2 infections (*n* = 327) were collected during no-restriction periods (white zone), and the 6.2% (*n* = 38) during lockdown (red zone). The remaining diagnosis were performed during light restriction periods (yellow and orange zone).

### Distribution of SARS-CoV-2 lineages affecting paediatric population

**Figure 1:**
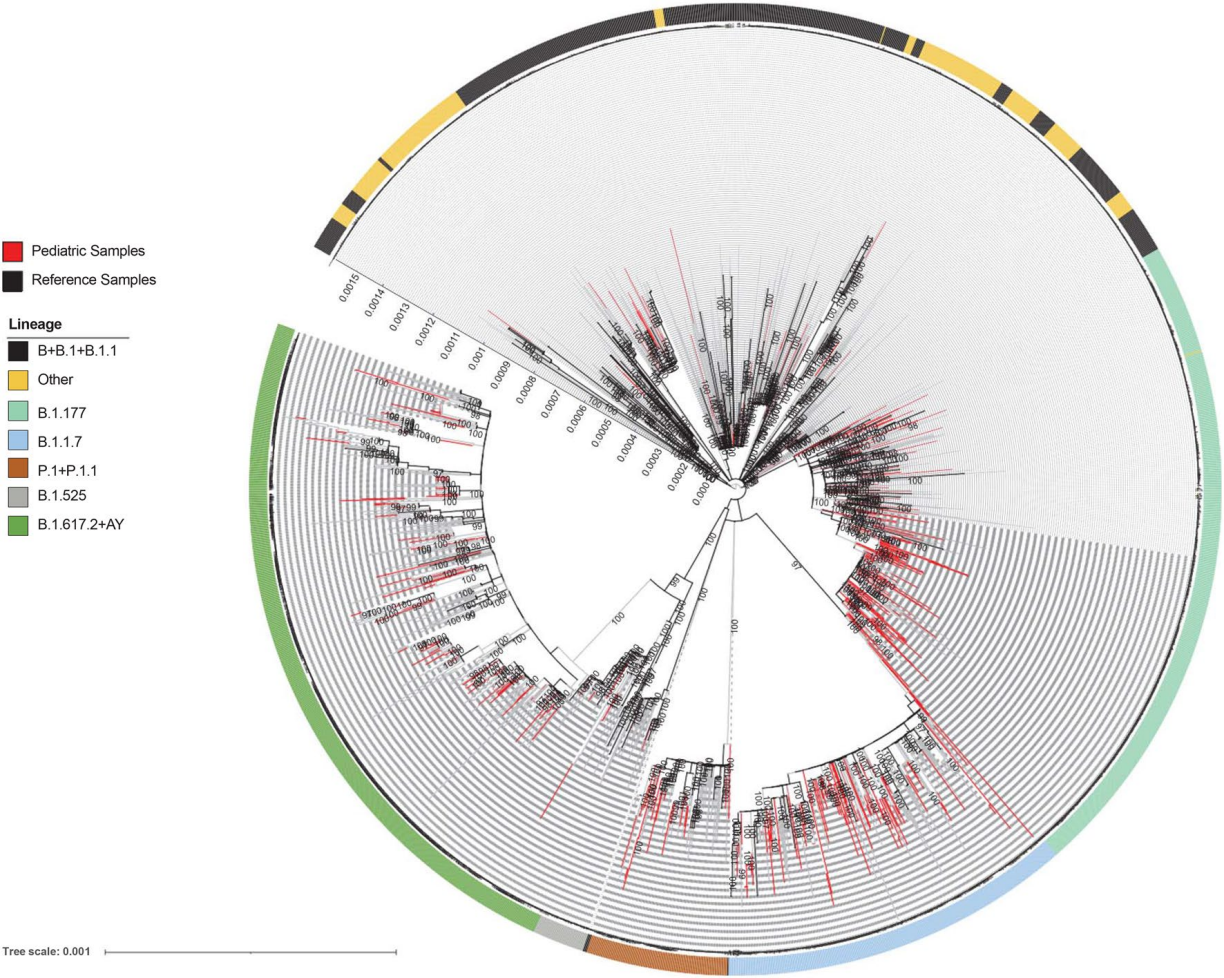
Estimated maximum likelihood phylogeny of SARS-CoV-2 genomes from population aged ≤ 12 years diagnosed at OPBG (*n* = 612, red taxa). Representative SARS-CoV-2 genomes retrieved by GISAID (*n* = 1233) and adolescent and adult SARS-CoV-2 infected population diagnosed in the same geographical area (*n* = 410) (gray taxa) were also included. The phylogeny was estimated with Iqtree with 1000 replicates fast bootstrapping. Major lineages were highlighted by black (B + B-1 + B.1.1), in light green (B.1.177), in light blue (B.1.1.7), in brown (P.1 and P.1.1), in gray (B.1.525), in dark green (B.1.617.2 + AY), and in yellow (other) circles.

**Figure 2:**
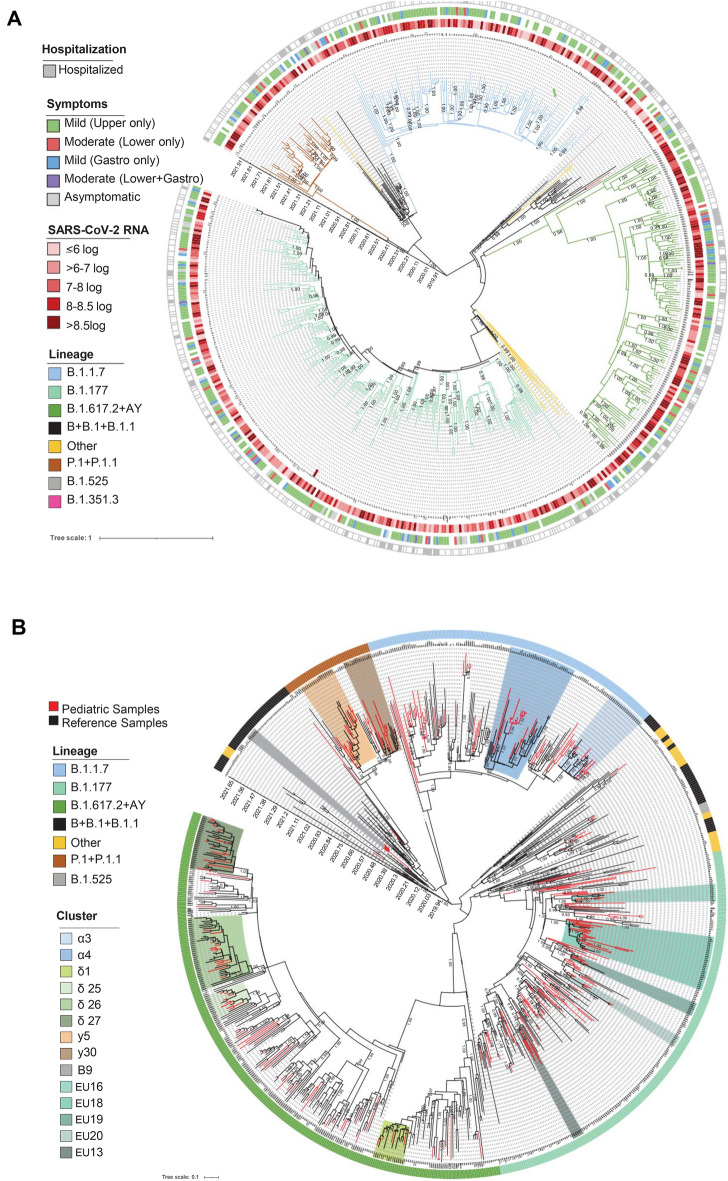
Bayesian phylogeographic reconstruction incorporating date of diagnosis of the 612 SARS-CoV-2 sequences obtained by population aged ≤ 12 years (**A**). SARS-CoV-2 genomes were highlighted in different colors against lineage. Information regarding Hospitalization, viral load and symptoms were also reported. Four independent chains were run for 50 million states. Parameters and trees were sampled every 1000 states. (**B**) Sampling of representative SARS-CoV-2 genomes retrieved by GISAID (*n* = 294), by adolescent and adult SARS-CoV-2 infected population diagnosed in the same geographical area (*n* = 207) (gray taxa), and by population aged ≤ 12 (*N* = 318) were included. Two independent chains were run for 50 million states. Parameters and trees were sampled every 1,000 states. Local clusters of SARS-CoV-2 sequences supported by an intra-genetic distance < 0.0002 and a posterior probability ≥ 0.99 were highlighted in different colors.

The distribution of sequences sampled in children up to the end of August 2021 against clinical characteristics and against SARS-CoV-2 global context are shown by a ML tree in Fig. 1 and by time-scale phylogeny in Fig. 2A, according to PANGOLIN application^22^. Demographic and clinical characteristics of patients infected with SARS-CoV-2 against lineages are reported in Table 1.

Most of SARS-CoV-2 infections (*n* = 253, 41.3%) belonged to lineage B.1.177 (EU) and affected children with median age 2 (IQR: 1–6) years between October 2020 and January 2021. B.1.177 sequences mainly belonged to 20E (EU1) lineage (*n* = 250, 98.8%) and were characterized by S:C22227T(A222V), and N:C28932T(A220V)^31^.

B.1.617.2 and AY sublineages were found in 139 (22.7%) patients with median age 2 (IQR: 1–7) years between July and August 2021, followed by B.1.1.7 (alpha clade) found in 127 (20.8%) patients aged median 3 (1–6) years between March and April 2021. As the end of August 2021, AY.43 (*n* = 51, 36.7% of sampled sequences) and AY.39 (*n* = 32, 23.0% of sampled sequences) were the most common delta clade sublineages detected in paediatric population.

The P.1 and P.1.1 (gamma clade) were found only in 35 individuals with median age 1 (IQR: 1–6) year and diagnosed between March and May 2021. Of note, 11 (31.4%) patients were of foreign origin, mainly from Southern Est Europe. The two sublineages differed for a single nucleotide polymorphism in RdRp:C13720T(P85S).

The B/B.1/B1.1 lineages characterizing the initial months of the SARS-CoV-2 pandemic were present only in 30 individuals aged 5 (1–8) years diagnosed between March and October 2020. This low number of B-related infections could be explained by the low number of children diagnosed as SARS-CoV-2 positive during the early phase of the pandemic (Supplementary Fig. 3).

Other lineages were detected in 28 patients and involved among others the B.1.160 (*n* = 14), the B.1.525 (*n* = 2), and the variant of concern B.1.351 (*n* = 1). Of note, the B.1.160 (20A.EU2) was found in all European recipients, 5 of them belonging to Southern East Europe. This lineage became common in Europe after summer 2020 and was characterized by the S: G22992A(S477N), known to strengthen the binding of the SARS-COV-2 spike with the human ACE2 receptor^32^.

The composition of sequences did not change substantially from SARS-CoV-2 sequences retrieved from general population of the same geographical origin and from GISAID (Fig. 1). Concordant with the lineages’ modification over time, the genetic pairwise distance of the 612 sequences indicated that the SARS-CoV-2 sequences evolved progressively during time (rho = 0.682, Supplementary Fig. 2).

Polymorphisms commonly shared in all lineages (prevalence > 90.0%) were the NSP3:synC3037T, the RdRp:C14408T(P314L) and the S:A23403G(D614G). As expected, N:G28881T(R203K) and N:G28883C(G204R), known to increase transmissibility potential of SARS-CoV-2^33^, were detected in B/B.1/B.1.1 (prevalence 73.0% and 76.7%, respectively), B.1.1.7 (prevalence 95.3% both), P.1/P.1.1 (prevalence 100.0% both), B.1.525 and B.1.351 lineages. As expected, and as previously reported in adult population^34,35^, gamma and delta clades were characterized by the highest viral load at diagnosis (SARS-CoV-2 RNA log copies/mL: 8.0 [6.1–8.6] and 8.4 [2.3–9.8], respectively) (Table 1 and Fig. 2A).

No significant association was found between lineages and COVID-19 clinical presentation, even if a low number of moderate/severe manifestations was found in presence of B.1.1.7 lineage (Table 1 and Fig. 2A).

### Evidence of local transmission clusters

By looking at the time-scale phylogeny, it was possible to identify clear transmission chains and clusters, and to clarify the dynamic of viral lineages in the paediatric population (Fig. 2B). The characteristics of the local clusters, with insights for clusters composed of ≥ 10 sequences were reported in Supplementary Table 2.

Overall, 129 sequences (21.1% of total paediatric sequences) were found in clusters, six of them composed of ≥ 10 sequences.

Lineage B.1.1 was characterized by limited local transmission, probably due to the low number of SARS-CoV-2 diagnoses in children during the first months of the pandemic. The only one local cluster, cluster B9, was characterized by a posterior probability of 1.00 and a tMRCA dated June, 8 2020 (May, 24-June, 13). It was composed of 6 sequences from children almost exclusively aged less than 2 years (except for one 4-year-old child). Five out of six children had a SARS-CoV-2 load in nasopharyngeal swabs > 7.0 log copies/mL. All children lived in Rome, but two of them had a Nigerian origin. Of note, a sequence diagnosed in South Africa on March 31 2020, is at the origin of this cluster, confirming the probable foreign origin of this cluster. Two children experienced a SARS-CoV-2 related pneumonia. All cluster B9 sequences were characterized by the NSP3:synC7639T.

Lineage B.1.177 was characterized by 5 local clusters, involving 44 sequences (17.4% of B.1.177 sequences). Among these 5 clusters, EU18 (posterior probability = 1.00) was composed of 24 sequences of paediatric patients, all Italian and residing in Rome, except for two of South American and Southern East Europe origin, respectively. Only four sequences belonged to adult individuals (age range: 22–61 years), supposing a sustained transmission among children aged less than 5 (representing the 80.6% of total paediatric clustering sequences, Supplementary Table 2) probably started in the middle/late August 2020. Cluster EU18 sequences were characterized by the NSP3:A6183G(K1155R), the NSP6:synT11836C, the RdRp:G15438T(M657I), N:synG229254A/T and N:C28706T(H145Y).

Two transmission chains probably starting between January and February 2021 were detected within P.1.1 lineage (Cluster γ5 and γ30, Fig. 2B and Supplementary Table 2). While cluster γ30 contains only 4 paediatric sequences intermixed with adult and global SARS-CoV-2 sequences, cluster γ5 involved a tracing network of 18 individuals, 12 of them (66.7%) below 12-years of age. All patients were diagnosed in Rome, but four of them had foreign origin. The earliest and most closely related strain was a sequence from a 4-months old child of Southern East Europe origin collected in late April 2021 in Rome, confirming a multi-seeded transmission. In line with this, the most recent ancestor of this cluster dates to February 12, 2021 (Feb 8 – Feb 24). All sequences were characterized by the RdRp:C13720T(P85S), NSP2:C2445T(T547I) and NSP4:synC9565T.

Twenty-two sequences from paediatric patients composed two main chains of lineage B.1.1.7 (posterior probability = 1, Fig. 2B, Supplementary Table 2). Cluster α4 involved 10/11 paediatric patients aged 4 (IQR: < 1–5), who were infected in Lazio region (mainly in Rome), and diagnosed after March, 14 2021. Half patients had a Southern Europe origin, suggesting a potential epidemiological linkage with this part of Europe. The most recent ancestor of this cluster dates back to February 17, 2021 (January 31-March 7). All patients experienced a mild infection, and exclusively reported upper respiratory airways symptoms (9/9 with information available). All sequences were characterized by the NSP8:C12525T(T145I), NSP14:synT18069C, ORF3a:synC25603T, ORF3a:C26110T(P240S), ORF8:C28087T(A65V), and N:synG29179T.

The other chain (Cluster α3) was composed of a total of 21 sequences (12 [57.1%] from paediatric individuals), all of them characterized by the ORF8:T28245G(L118V), ORF8:ins282458CTG(ins119L), NSP2:A1643T(N280Y). Sequences from paediatric patients were intermixed with sequences from adult patients (age range 32–77), diagnosed in Rome in the same period (Fig. 2B). Paediatric patients, with a median age of 1 year (IQR: < 1–2), were most Caucasian (11, 91.7%), infected in Lazio region (mainly in Rome) and diagnosed between March/April 2021 (Supplementary Table 2). Mild symptoms (mainly upper respiratory) were the most frequently reported (9/10 with information available).

Delta clade was characterized by 4 local clusters, involving 39 sequences (28.1% of delta clade sequences). The high prevalence of delta sequences in local clusters suggests the sustained circulation in the paediatric population of this clade since its emergence. Among these 4 clusters, δ26 (posterior probability = 1.00) was composed of 37 sequences, 14 of them (37.8%) paediatrics and with a SARS-CoV-2 load in nasopharyngeal swabs > 7.0 log copies/mL in 12/14 patients (Supplementary Table 2). All patients with exception for one are Caucasian. Sequences were characterized by the NSP1:synC745T, NSP3:synC6730T, RdRp:synC13944T and RdRp:G15906T(Q813H), ORF3a:G25471(D27Y), and M:synA26786G. As of 3 December 2021, all these sequences were defined as AY.125, supporting the hypothesis of a delta-subclade originated in late May, as defined by the tMRCA (May 12, 2021 [April 27-May 19]), and expanded in paediatric and adult Italian population in central Italy in the middle of July 2021.

Cluster δ27 (posterior probability = 1.00), probably originated in the late May/early June 2021 was composed of 28 sequences, 13 of them (46.7%) belonged to paediatric individuals and with a SARS-CoV-2 load in nasopharyngeal swabs > 8.0 log copies/mL in 12/13 patients (Supplementary Table 2). The remaining individuals belonged to adolescent (*n* = 2, aged 15 and 18 years) and adult population (*n* = 11, age range: 20–85 years). Sequences were characterized by the RdRp:synC16111T, the NSP13:G17671A (V479I) and the S:G23593C(G677H), reported as recurrent arising independently in many SARS-CoV-2 lineages circulating worldwide by the end of 2020, and known to enhance viral infectivity and neutralizing antibodies resistance^36,37^.

Univariate and multivariate logistic regression models were performed to identify potential factors associated with local clusters (Table 2). The results showed that in our paediatric population, patients aged ≥ 5 were less commonly found in clusters (adjusted odds ratio, AOR [95% CI]: 0.49 [0.29–0.84]; *P* = 0.009), whereas there was a positive association between the presence in clusters and individuals infected with a delta or gamma clade (AOR [95% CI]: 1.89 [1.18–3.03] and 4.05 [1.88–8.75]; *P* = 0.008 and *P* < 0.0001). No associations were found with COVID-19 presentation. By considering the timing of diagnosis, no significant association with lockdown periods was detected.

**Table 2:** Multivariable logistic regression analysis of factors associated with local transmission clusters.

Variable associated to local clusters	Univariable analysis		Multivariable analysis	
	OR (95% CI)	*P*-Value	AOR (95% CI)	*P*-Value
Gender (male vs. female)	0.98 (0.66–1.45)	0.922		0.996
**Age**				
< 1	1.12 (0.75–1.68)	0.578		0.335
1–5	1.47 (0.99–2.19)	0.056		0.335
≥ 5	0.54 (0.34–0.86)	0.010	0.49 (0.29–0.84)	0.009
Origin (Caucasian vs. non-caucasian)	0.79 (0.46–1.34)	0.378		0.177
Residency (Lazio vs. Out of Lazio)	1.13 (0.42–3.05)	0.813		0.886
**Lineage**				
B/B.1/B.1.1	0.93 (0.37–2.33)	0.882		0.617
B.1.177—EU	0.68 (0.45–1.02)	0.061		0.904
B.1.1.7—alpha	0.74 (0.45–1.23)	0.245		0.870
P.1/P.1.1—gamma	4.45 (2.22–8.90)	< 0.0001	4.05 (1.88–8.75)	< 0.0001
B.1.617.2 + AY—delta	1.66 (1.07–2.57)	0.023	1.89 (1.18–3.03)	0.008
Other^a^	0.0 (0.0- inf)	0.998		0.998
Hospitalization	0.97 (0.58–1.61)	0.905		0.591
SARS-CoV-2 RNA ddPCR (copies/mL)	1.23 (1.06–1.43)	0.008		0.192
**COVID-19 at SARS-CoV2- testing**				
Mild^b^	1.27 (0.80–2.02)	0.318		0.490
Moderate/Severe^c^	1.31 (0.68–2.54)	0.417		0.379
Asymtomatic	0.77 (0.33–1.81)	0.551		0.967
Comorbidity	0.79 (0.37–1.66)	0.531		0.660
**Lockdown**				
Red zone	0.69 (0.28–1.68)	0.412		0.996
Orange zone	1.10 (0.61–2.00)	0.749		0.804
Yellow zone	1.00 (0.65–1.53)	0.983		0.589
White zone	1.04 (0.71–1.54)	0.831		0.727

*OR* odds ratio, *AOR* adjusted odds ratio, *CI* confidence interval. ^a^Includes: B.1.160 (*n* = 14), B.1.221 (*n* = 2); B.1.258 (*n* = 4), B.1.351 (*n* = 1), B.1.36 (*n* = 2), B.1.525 (*n* = 2), B.1.398 (*n* = 1), B.1.416 (*n* = 1), C.36.3 (*n* = 1). ^be^Including: symptoms of upper respiratory airways (rinhitis, pharyngo-adenitis, laryngitis) and/or gastrointestinal symptoms. ^c^Including: symptoms of lower respiratory airways (pneumonia, bronchitis and bronchiolitis) with or without gastrointestinal symptoms.

### Correlation with hospitalization

Univariate and multivariate logistic regression models were performed to define if lineages or clusters can be potentially associated with hospitalization (Table 3). As confounding factors gender, age, nationality, residency, SARS-CoV-2 viral load, symptoms at diagnosis, and comorbidities were considered. The results showed that in our paediatric population, lineages and clusters were not associated with hospitalization, with the exception for B.1.1.7 lineage, significantly and negatively associated with hospitalization (AOR: 0.31 [0.13–0.71], *P* = 0.006). This lineage was also characterized by the lowest (even if not significant) number of COVID-19 moderate or severe manifestations (*n* = 4, 4.0%) compared with other SARS-CoV-2 lineages (Table 1).

**Table 3:** Multivariable logistic regression analysis of factors associated with Hospitalization.

Variable associated to Hospitalization	Univariable analysis		Multivariable analysis	
	OR (95% CI)	*P*-Value	OR (95% CI)	*P*-Value
Gender (male vs. female)	1.4 (0.95–2.21)	0.088	1.94 (1.16–3.26)	0.012
**Age**				
< *1*	2.41 (1.57–3.70)	< 0.0001	3.93 (2.26–6.82)	< 0.0001
1–5	0.50 (0.31–0.82)	0.006		0.967
> 5	0.70 (0.43–1.15)	0.161		0.967
Origin (Caucasian vs. non-caucasian)	0.45 (0.26–0.78)	0.004	0.36 (0.19–0.67)	0.002
Residency (Lazio vs. Out of Lazio)	0.52 (0.19–1.41)	0.201		0.784
**Lineage**				
B/B.1/B.1.1	3.08 (1.42–6.66)	0.004		0.055
B.1.177	0.89 (0.58–1.38)	0.608		0.111
B.1.1.7	0.29 (0.13–0.61)	0.001	0.31 (0.13–0.71)	0.006
P.1/P.1.1	0.93 (0.37–2.31)	0.868		0.743
B.1.617.2 + AY	1.67 (1.05–2.66)	0.031		0.311
Other^a^	1.16 (0.37–3.59)	0.801		0.689
In cluster	0.97 (0.58–1.61)	0.905		0.450
SARS-CoV-2 RNA ddPCR (copies/mL)	0.88 (0.75–1.03)	0.108		0.473
**COVID-19 at SARS-CoV-2 testing**				
Mild^b^	0.26 (0.16–0.43)	< 0.0001		0.044
Moderate/Severe^c^	12.18 (6.41–23.16)	< 0.0001	13.92 (6.66–29.07)	< 0.0001
Asymtomatic	0.21 (0.05–0.88)	0.033		0.058
Comorbidity	5.17 (2.81–9.50)	< 0.0001	10.73 (4.79–24.04)	< 0.0001

^a^Includes: B.1.160 (*n* = 14), B.1.221 (*n* = 2); B.1.258 (*n* = 4), B.1.351 (*n* = 1), B.1.36 (*n* = 2), B.1.525 (*n* = 2), B.1.398 (*n* = 1), B.1.416 (*n* = 1), C.36.3 (*n* = 1). ^b^Including: symptoms of upper respiratory airways (rinhitis, pharyngo-adenitis, laryngitis) and/or gastrointestinal symptoms. ^c^Including: symptoms of lower respiratory airways (pneumonia, bronchitis and bronchiolitis) with or without gastrointestinal symptoms.

As expected, patients aged < 1, patients with comorbidities, and moderate/severe COVID-19 were more frequently associated with hospitalization (*P* < 0.0001, Table 3).

## Discussion

These data, based on the genetic characterization of the SARS-CoV-2 strains circulating in paediatric population, implemented with demographics and clinical information, indicate that at least four lineages widely circulated in paediatric population over the four COVID-19 waves. Children were also part of SARS-CoV-2 community transmission, as supported by the evidence of 6 transmission chains composed of more than 10 paediatric SARS-CoV-2 sequences and characterized by fixed synonymous and non-synonymous substitutions. The 21.1% of SARS-CoV-2 sequences were indeed involved in local clusters, and 64.3% of them takes part in 6 large transmission chains.

While transmission dynamics were deeply investigated in general population, helping in defining the geographical mapping, tracking and evolution of SARS-CoV-2^20,31,34,38^, limited evidence relating to SARS-CoV-2 transmission dynamics and correlation with demographic and clinical characteristics are available so far in paediatric population. The relationship between age, viral load, lineages and transmission trajectories across the full symptom spectrum of SARS-CoV-2 infection has not been comprehensively investigated. The role of different ages in transmitting the virus is also uncertain.

In our study, the SARS-CoV-2 positivity rate in children aged ≤ 12 years increased from 0.6% between March and July, 2020 to 5.2% between August and December 2020 remaining almost stable since August 2021. This positivity ranges should be interpreted in the context of circulating variants. In the early phase of the pandemic, the low number of children diagnosed as SARS-CoV-2 positive^39^ were infected by B/B.1/B1.1 lineages. These lineages were still characterized by local transmission, as described by the B9 cluster, emerging in late May/early June 2020, and composed of 6 sequences from children exclusively aged less than 5 years and with a SARS-CoV-2 load in nasopharynx > 7.0 log copies/mL. The presence of a local transmission cluster composed of children just three months after the start of Italian SARS-CoV-2 epidemic^40^ can be interpreted as the first evidence of the active role of children in the community SARS-CoV-2 transmission dynamics. Lineage B.1.177 was found to predominate between October 2020 and January 2021 and accounted for the 20.8% of all SARS-CoV-2 sequences analyzed in this study. Sequences of B.1.177 lineage belonged to 20E (EU1) clade^31^, whose earliest sequences were found in samples collected on June 20, 2020, in Spain and in the Netherlands. By the end of August, 20E (EU1) sequences had also been detected in most of European countries, including Italy^31^. The 17.4% of 20E sequences circulating in children were also involved in local transmission clusters, one of them composed of 24 sequences characterized by 5 SNPs. Some of these SNPs were previously described in Northern Europe^41^, and as of 3 December 2021 were not related with transmission or pathogenicity alterations. Among the variants rapidly causing concerns, P.1 (gamma clade), B.1.1.7 (alpha clade), and B.1.617 (delta clade) raised in paediatric population between March and July 2021. All these variants were clearly characterized by an increased transmissibility compared with the previous wildtype lineages^34,42,43^. In line with this, B.1.1.7 and B.1.617.2 + AY were the most prevalent variants in our population after the B.1.177 (20.8% and 22.7% of the 612 SARS-CoV-2 sequences, respectively). Transmission clusters with evidence of expanded evolution were detected in both alpha and delta clades. While clusters of alpha clade involved 17.3% of total B.1.1.7 sequences and did not report evidences of genetic alterations able to increase transmission or pathogenicity, transmission chains of delta clade involved the 28.1% of delta sequences. Thirteen of them reported the Q677H mutation known to enhance viral infectivity and neutralizing antibodies resistance^36,37^. The rapid mechanism of delta clade adaptation, and its onward transmission in local paediatric population might explain its outcompeting and dominance respect pre- and co-existing lineages as P.1 and B.1.1.7, as previously observed in adult population^44^. As of 3 December 2021, delta clade remains the dominant lineage in the paediatric population in Lazio region. The SARS-CoV-2 positivity rate in paediatric population diagnosed in our center between September and November, 2021 was 3.4%, almost stable respect June and August 2021. No B.1.1.529 variant (omicron clade) was detected.

Differently by alpha and delta clade, a limited spread of gamma clade was observed. Only 35 sequences (5.7% of the whole population here analysed) mainly concentrated between March and May 2021 were indeed identified. Of note, 22 of these 35 sequences were involved in probable transmission patterns. The limited spread and the closely relatedness of the P.1 variants detected in paediatric population might reflect the appropriate control strategy against this variant at local level. The P.1 limited spread in paediatric population can also reflect the rapid dominance of delta clade respect to this co-existing lineage, as previously stated.

Looking at factors associated with local transmission clusters, multivariate logistic regression model identified both gamma and delta clades as positively associated with transmission chains, implying that local transmission dynamics were the key drivers of the spread of these lineages in the paediatric population. Multivariate model also defines that patients aged less than 5 years were more frequently found in clusters respect to patients aged > 5 years (Table 2). Patients aged < 5 years were also characterized by a higher SARS-CoV-2 viral load in the nasopharynx respect to older paediatric population (SARS-CoV-2 log copies/mL: 8.3 [6.3–8.6] in > 5-year-old patients vs. 7.7 [6.2–8.4] in 1–5-year-old patients vs. 7.1 [6.0–8.3] in < 1-year-old patients. P < 0.0001), thus supporting their role as common SARS-CoV-2 transmitters. Thus, our study demonstrates that the role of children in SARS-CoV-2 community transmission should not be underestimated and strengthen the vaccine EMA recommendation approval of SARS-CoV-2 vaccine for children aged 5–11^45^. Vaccination optimization models including paediatric population is worthwhile to reduce the burden of hospitalization and risk of severe manifestations in this young category of patients, but may also increase the chance of reducing SARS-CoV-2 circulation, and thus the risk of new spreading variants. Individuals aged < 5 years, mainly associated with local transmission clusters, will be the latest group to benefit of SARS-CoV-2 vaccination. Clinical trials are to date ongoing to determine the optimal dose of vaccines to protect this group of children while minimizing potential adverse events. These findings advocate for a rapid finalization of vaccine in this age group. Meanwhile, the continued molecular surveillance in this unvaccinated population remains crucial.

The effort to implement with demographic and clinical data the 612 SARS-CoV-2 sequences obtained by paediatric individuals made it possible to estimate the role of SARS-CoV-2 variability in transmission dynamic, SARS-CoV-2 presentation and hospitalization. According to other reports^46^ most of the paediatric patients presented with mild disease (83.2%), while moderate conditions accounted for the 9.7% of cases. No significant association was found between lineages and COVID-19 presentation, even if a lower number of moderate/severe cases were found during alpha-clade epidemic (Table 1). In line with this, patients involved in clusters of B.1.1.7 frequently reported only mild symptoms (moderate/sever infection was detected only in one in-cluster case), and no new amino acid mutation was detected in spike or nucleocapsid proteins, both implicated in SARS-CoV-2 virulence and pathogenicity^32,33,36,37^. B.1.1.7 variant was also found negatively associated with hospitalization in our multivariate model (Table 3). These results confirmed evidence observed in adult population, affirming that no change in symptoms or their duration were associated with alpha clade^47,48^.

We acknowledge some limitations to this study. To warrant high quality sequences and good genomic coverage, only samples with Ct values ≤ 29 were selected (see Supplementary Results). In order to exclude sampling bias that could affect viral diversity driven by this selection, the characteristics of the general paediatric SARS-CoV-2 affected population were compared with those of sampled population and no major differences were highlighted (Supplementary Table 1). Limitations to the assessments of the proportions of asymptomatic cases should be noted: most of our paediatric population get tested only when children have symptoms, so relatively few asymptomatic infections are recorded. Our study doesn’t assess the on-going clinical symptoms neither their persistence after SARS-CoV-2 clearance. The paediatric nature of this study, and the paediatric Hospital in which sequences were collected, have limited the possibility to include adult patients with direct epidemiological linkage with the children here described, thus affecting the possibility to define household transmission trajectories. This limitation can also represent a strength, because the presence of adult individuals not sharing contacts with paediatric patients has confirmed the role of younger population in SARS-CoV-2 transmission at community level.

In conclusion, we report unique and updated information on the temporal trends of SARS-CoV-2 variants circulating in paediatric population, and their association with demographic and clinical manifestations. The data provided increase knowledge of SARS-CoV-2 transmission dynamic and the role of children in the community transmission. Transmission chains involved children mainly aged < 5 years and were detected in all phases of the pandemic. The frequent copresence of sequences from adults with no epidemiological link according to demographic data suggests both the superimposable distribution of paediatric and global SARS-CoV-2 epidemics, and the active role of paediatric population in SARS-CoV-2 community transmission. Even if children and adolescents usually demonstrate fewer and milder symptoms of SARS-CoV-2 infection compared to adults and are less likely than adults to experience severe COVID-19, continued molecular surveillance in this unvaccinated population will be essential to prevent hospitalization risk, COVID-19 related sequelae, SARS-CoV-2 transmission and spread, and thus new variants’ selection.

## Supplementary Information

Supplementary Information.

## Data Availability

The 1022 SARS-CoV-2 sequences obtained in this study are openly available on GISAID portal and European Nucleotide Archive under the accession numbers EPI_ISL_7671548-EPI_ISL_7694367 and ERS8842923-ERS8843944, respectively. The list of accession numbers is available in the Supplementary Data 3. The list of the whole-genome SARS-CoV-2 sequences (*n* = 3244) retrieved from GISAID (gisaid.org) on 18 September 2021 and their corresponding accession numbers and lineages are available in the Supplementary Data 1. The list of the significant homoplastic positions identified by HomoplasyFinder and TreeTime are available in the Supplementary Data 2. Information regarding SARS-CoV-2 incidence rate in population aged ≤ 14 was made available on ECDC web page (https://www.ecdc.europa.eu/en/publications-data/covid-19-data-14-day-age-notification-rate-new-cases). The study protocol was approved by local Research Ethics Committee of OPBG (prot. 2384_OPBG_2021). This study was conducted in accordance with the principles of the 1964 Declaration of Helsinki. Informed consent was waived in accordance with the hospital regulations on observational retrospective studies. The de-identified data regarding demographic and clinical features related to each patient are available on reasonable request from the corresponding author. Data are not publicly available in order to fully comply to privacy guarantee.

## Code availability

Beast.xml files used to infer the corresponding time-scaled maximum clade credibility tree and the Bayesian tree interference were available at 10.5281/zenodo.5840191.
