# Supplementary material for: Epidemiological characterization of SARS-CoV-2 variants in children over the four COVID-19 waves and correlation with clinical presentation
Source: Sci Rep. 2022 Jun 17;12:10194. doi: 10.1038/s41598-022-14426-0 (PMC9204374; doi:10.1038/s41598-022-14426-0)
Supplement: Supplementary file 1 — Supplementary Information. [file 41598_2022_14426_MOESM1_ESM.pdf]

Supplementary Information

Title: Epidemiological characterization of SARS-CoV-2 variants in children over the four COVID-19 waves and correlation with clinical presentation

Running head: SARS-CoV-2 variants and COVID-19 presentation in children

Claudia Alteri[1,2], Rossana Scutari[1,2§], Valentino Costabile[1,2§], Luna Colagrossi[1], Katia Yu La Rosa[1], Emanuele Agolini[1], Valentina Lanari[1], Sara Chiurchiù[3], Lorenza Romani[3], Anna Markowic[3], Paola Bernaschi[3], Cristina Russo[3], Antonio Novelli[1], Stefania Bernardi[4], Andrea Campana[4], Antonio Villani[4], Carlo Federico Perno[1,3✉]

[1]Multimodal Research Area, Bambino Gesù Children Hospital IRCCS, Rome, Italy

[2]Department of Oncology and Hemato-Oncology, University of Milan, Milan, Italy

[3]Microbiology and Diagnostics in Immunology Unit, Bambino Gesù Children Hospital IRCCS, Rome, Italy

[4]Academic Department of Pediatrics, Bambino Gesù Children's Hospital, IRCCS, Rome, Italy

[§] These authors contributed equally: RS, VC

[✉]email: carlofederico.perno@opbg.net

Supplementary Results

Sampling criteria and patients' characteristics

From March 05, 2020, through August 31, 2021, nasopharyngeal swabs taken from a total of 45,573 individuals aged ≤12 years were screened for SARS-CoV-2 infection at the main pediatric Hospital in Rome (Supplementary Table 1). A diagnosis of COVID-19 was made for 2,399 of them. For 1,988 patients we were able to retrieve information regarding sex, origin, and exact age. To warrant high quality sequences and good genomic coverage, samples with Ct values >29 or Ct values not easily retrieved (n=1,006) were excluded. Out of the remaining 982 patients, 731 samples were selected for inclusion, according to the samples' availability. The sampling criteria of the 731 samples was provided by Supplementary Figure 2. In Supplementary Table 1, the characteristics of the 1,988 SARS-CoV-2 infected paediatric patients with sex, and age information, were compared with the 731 selected samples. Likelihood Ratio Test, followed by a multinomial logistic regression model to estimate 95% confidence intervals of odds ratios, was used to compare demographic and clinical findings between general and selected SARS-CoV-2 paediatric infected populations. By looking at residency, origin, age and sex, the selected population is well representative of SARS-CoV-2 infected paediatric population. Prevalence of population aged <1 year and prevalence of patients with Latin American origin are slightly higher in the selected population compared to the general population (35.0% vs. 26.7%, P<0.001; and 3.1% vs. 2.0%, P=0.021). Symptoms were largely comparable, even though a higher prevalence of asymptomatic individuals were observed in the selected population compared to the general population (8.4% vs 12.3%, P<0.001). Hospitalizations were also comparable (18.9% in the general population vs. 20.4% in the selected population, P=0.272).

The SARS-CoV-2 full length genome sequencing performed in our hospital was largely superimposable to SARS-CoV-2 incidence rate in the population aged ≤14 in Italy (Supplementary Fig.3, panel A) and to SARS-CoV-2 diagnoses with CT<29 in population aged ≤12 at OPBG (Supplementary Fig.3, panel B), even though a lower coverage was observed between October and November 2020.

Supplementary Table 1. **Demographic, and clinical findings of general SARS-CoV-2 infected paediatric population and the 731 originally selected SARS-CoV-2 infected paediatric population.**

	General COVID-19 affected population, N=1,988	Sampled population for SARS-CoV-2 sequencing, N=731	Odds Ratio (Confidence Interval)[§]	P-value[§]
**Demographics and clinical characteristics**				
Age, years:				
<1	530 (26.7)	256 (35.0)	2.03 (1.60-2.56)	<0.0001
1-5	770 (38.7)	258 (35.3)	1.1 (0.88-1.36)	0.424
≥5	688 (34.6)	217 (29.7)	0.92 (0.74-1.15)	0.453
Sex, Male	1074 (54.0)	416 (56.9)	1.18 (0.98-1.42)	0.083
Origin				
Caucasian	1748 (87.9)	624 (85.4)	1.27 (0.67-2.40)	0.463
Asian	90 (4.5)	40 (5.5)	1.83 (0.86-3.88)	0.116
North American	46 (2.3)	14 (1.9)	0.75 (0.40-1.41)	0.369
Latin American	40 (2.0)	23 (3.1)	2.87 (1.17-7.07)	0.021
African	64 (3.2)	30 (4.1)	2.02 (0.91-4.48)	0.085
Residency:				
Lazio	1759 (88.5)	646 (88.4)	0.98 (0.74-1.31)	0.908
Others[a]/Unknown	229 (11.5)	85 (11.6)	-	-
COVID-19 at SARS-CoV-2 testing[b]:				
Mild[c]	925 (80.1)	470 (82.5)	0.72 (0.46-1.12)	0.139
Moderate/Severe[d]	88 (7.6)	52 (9.1)	1.53 (0.98-2.38)	0.059
Asymtomatic	142 (12.3)	48 (8.4)	0.35 (0.20-0.61)	<0.0001
Hospitalization[e]	232 (18.9)	120 (20.4)	1.21 (0.91-1.61)	0.198
SARS-CoV-2 rtPCR[f]:				
Mean cycle thresholds	27 (19-34)	18 (15-23)	37.29 (16.33-85.16)	<0.0001
E	25 (18-34)	18 (15-23)	0.42 (0.29-0.62)	<0.0001
RdRp/S	27 (19-34)	19 (16-23)	0.20 (0.13-1.029)	<0.0001
N	26 (18-33)	18 (15-23)	0.42 (0.34-0.51)	<0.0001

Data are expressed as median (IQR), or N (%). [§]For comparisons of demographic and clinical findings between general and selected SARS-CoV-2 infected populations, a Likelihood Ratio Test followed by a multinomial logistic regression model to estimate 95% confidence intervals of odds ratios was used. Two-sided P-values are reported. [a]Others include: Abruzzo (n=9), Calabria (n=6), Campania (n=16), Emilia-Romagna (n=3), Lombardia (n=9), Marche (n=3), Molise (n=, 1), Puglia (n=15), Sardegna (n=2), Sicilia (n=3), Toscana (n=3), Umbria (n=2), Veneto (n=1); Unknown (n=213). [b]Data available for 1155 patients. [c]Including: symptoms of upper respiratory airways (rinhitis, pharyngo-adenitis, laryngitis) and/or gastrointestinal symptoms. [d]Including: symptoms of lower respiratory airways (pneumonia, bronchitis and bronchiolitis) with or without gastrointestinal symptoms. [e]Data available for 763 patients. [f]Real-time reverse transcription PCR Ct (cycle threshold) values of these samples ranged from 9 to 40 by real-time RT-PCR (AllplexTM 2019-nCoV Assay, Seegene; Xpert Xpress SARS-CoV-2 Assay, Cepheid; Simplexa® COVID-19 Direct Kit, DiaSorin Molecular).

Supplementary Table 2. **Demographic and clinical characteristics of local transmission clusters**

	**Out of clusters N=483**	**In clusters N=129**	**B.1.177 (Eu)**	**P.1 (Gamma)**	**B.1.1.7 (Alfa)**		**B.1.617.2/AY (Delta)**		**P-value[a]**	**P-value[b]**
			EU-18	γ-5	α-4	α-3	δ-26	δ-27		
Presence of patients aged ≤12 years	483	129	24	12	10	12	14	13	-	-
Presence of adolescents or adult patients (>12 years)	-	-	4	6	1	9	23	15	-	-
Demographic characteristics										
Age, years:										
<1	167 (34.6)	48 (37.2)	9 (37.5)	2 (16.7)	3 (30.0)	6 (50.0)	5 (38.7)	5 (38.5)	0.604	0.412
1-5	162 (33.5)	55 (42.6)	13 (54.2)	5 (41.7)	6 (60.0)	5 (41.7)	4 (28.6)	4 (30.8)	0.062	0.098
≥5	154 (31.9)	26 (20.2)	2 (8.3)	5 (41.7)	1 (10.0)	1 (8.3)	5 (35.7)	4 (30.8)	0.009	0.295
Sex, Male	271 (56.1)	73 (56.6)	12 (50.0)	7 (58.3)	8 (80.0)	9 (75.0)	8 (57.1)	6 (46.2)	1.0	0.181
Residency (Lazio vs. Out of Lazio)	462 (95.7)	124 (96.1)	23 (95.8)	12 (100.0)	9 (90.0)	11 (91.7)	14 (100.0)	13 (100.0)	1.0	0.803
Origin										
Caucasian	419 (86.7)	108 (83.7)	22 (91.7)	9 (75.0)	8 (80.0)	11 (91.7)	13 (92.8)	11 (84.6)	0.391	0.105
Asian	21 (4.3)	9 (7.0)	1 (4.2)	2 (16.7)	0 (0.0)	0 (0.0)	1 (7.1)	2 (15.4)	0.249	0.574
North American	8 (1.7)	2 (1.6)	0 (0.0)	0 (0.0)	0 (0.0)	0 (0.0)	0 (0.0)	0 (0.0)	1.000	0.876
Latin American	14 (2.9)	6 (4.7)	1 (4.2)	0 (0.0)	2 (20.0)	0 (0.0)	0 (0.0)	0 (0.0)	0.400	0.055
African	21 (4.3)	4 (3.1)	0 (0.0)	1 (8.3)	0 (0.0)	1 (8.3)	0 (0.0)	0 (0.0)	0.625	0.039
Clinical characteristics										
COVID-19 at SARS-CoV-2 testing[c]:										
Mild[d]	337 (83.4)	99 (82.5)	20 (87.0)	7 (70.0)	9 (100.0)	9 (90.0)	11 (78.6)	10 (83.3)	0.783	0.826
Moderate/Severe[e]	37 (9.2)	14 (11.7)	2 (8.7)	1 (10.0)	0 (0.0)	1 (10.0)	3 (21.1)	1 (8.3)	0.482	0.460
Asymtomatic	30 (7.4)	7 (5.8)	1 (4.3)	2 (20.0)	0 (0.0)	0 (0.0)	0 (0.0)	1 (8.3)	0.686	0.512
Comorbidity	42 (8.7)	9 (7.0)	0 (0.0)	2 (16.7)	1 (10.0)	2 (16.7)	0 (0.0)	1 (7.7)	0.595	0.122
Hospitalization, days[f]	83 (20.0)	24 (19.5)	3 (12.5)	2 (16.7)	0 (0.0)	3 (30.0)	3 (21.4)	3 (25.0)	1.000	0.964
Lenght of hospitalization[g]	4.7 (3.0-9.7)	4.0 (3.0-9.8)	6.0 (4.0-15.0)	7.1 (6.2-8.0)	-	5.0 (3.0-42)	2.0 (2.0-10.9)	3.0 (2.0-13.5)	0.970	0.390
SARS-CoV-2 RNA by ddPCR (log copies/mL)	7.7 (6.2-8.5)	7.7 (6.1-8.5)	7.6 (6.1-8.5)	6.9 (6.1-8.4)	8.0 (6.3-8.5)	6.8 (6.1-7.7)	8.5 (8.0-8.7)	8.5 (8.4-8.9)	0.798	0.03
≤ 6	93 (19.3)	13 (10.1)	4 (16.7)	1 (8.3)	0 (0.0)	2 (16.7)	0 (0.0)	0 (0.0)	0.013	0.967
> 6-7	95 (19.7)	29 (22.5)	7 (29.2)	5 (41.7)	3 (30.0)	1 (8.3)	2 (14.3)	1 (7.7)	0.463	0.599
>7-8	124 (25.7)	24 (18.6)	4 (16.7)	3 (25.0)	2 (20.0)	5 (41.7)	2 (14.3)	0 (0.0)	0.106	0.036
>8-8.5	76 (15.7)	24 (18.6)	3 (12.5)	0 (0.0)	2 (20.0)	1 (8.3)	4 (28.6)	4 (30.8)	0.424	0.303
> 8.5	95 (19.7)	39 (30.2)	6 (25.0)	3 (25.0)	3 (30.0)	3 (25.0)	6 (42.9)	8 (61.5)	0.012	0.176

Data are expressed as median (IQR), or N (%). [a]Two-sided P-values were calculated by Mann-Whitney test, or Fisher exact test, as appropriate in order to define differences between in and out clusters. [b]Two-sided P-values were calculated by Kruskal–Wallis test, or Chi-square test for trend, as appropriate, in order to define differences among clusters. [c]Data available for 524 patients. [d]Including: symptoms of upper respiratory airways (rinhitis, pharyngo-adenitis, laryngitis) and/or gastrointestinal symptoms. [e]Including: symptoms of lower respiratory airways (pneumonia, bronchitis and bronchiolitis) with or without gastrointestinal symptoms. [f]Data available for 536 patients. [g]Data available for 122 patients.

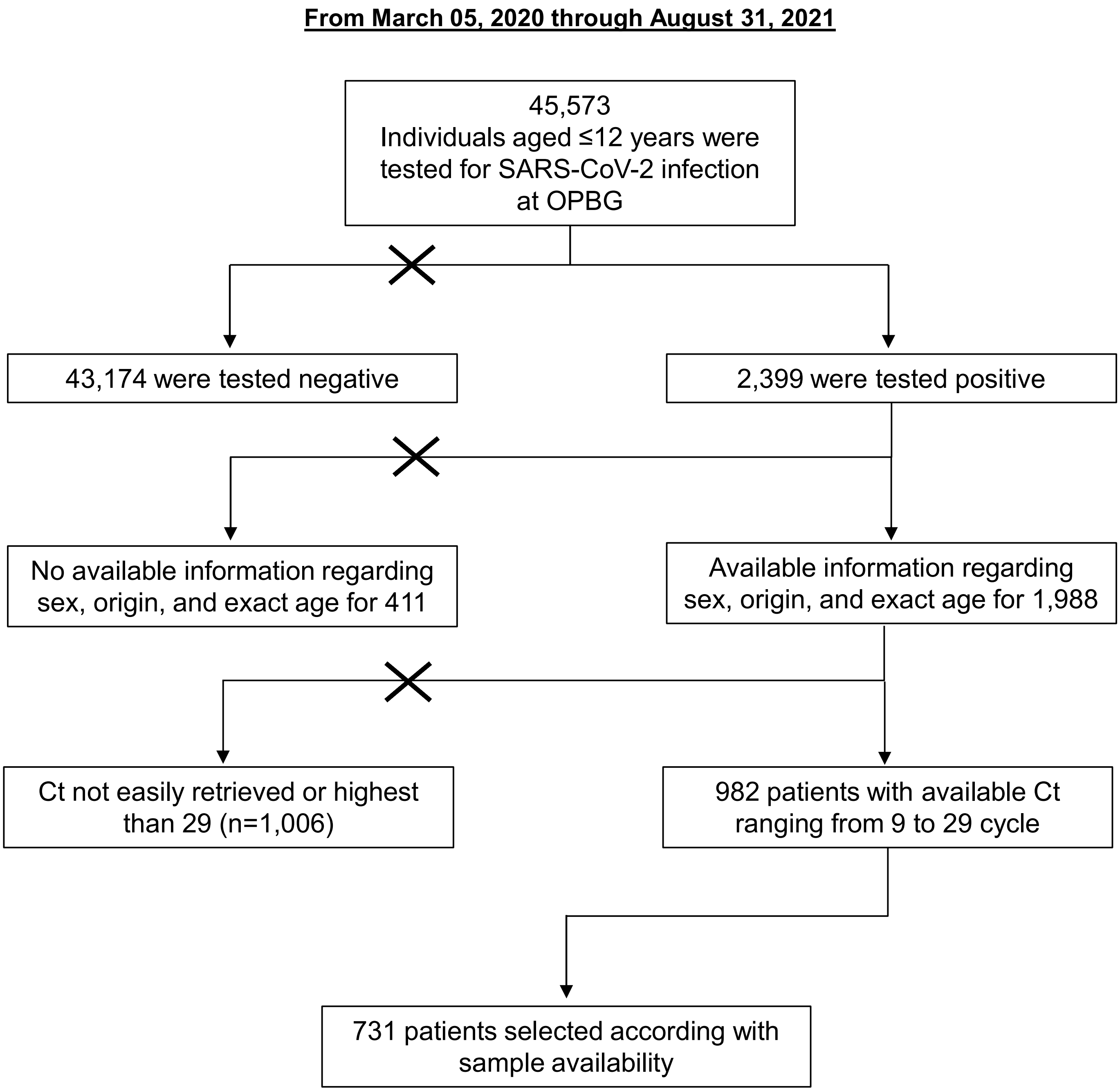

Supplementary Fig. 1. **Selection criteria for the 731 swab samples originally included in the study.** Ct: Cycle threshold

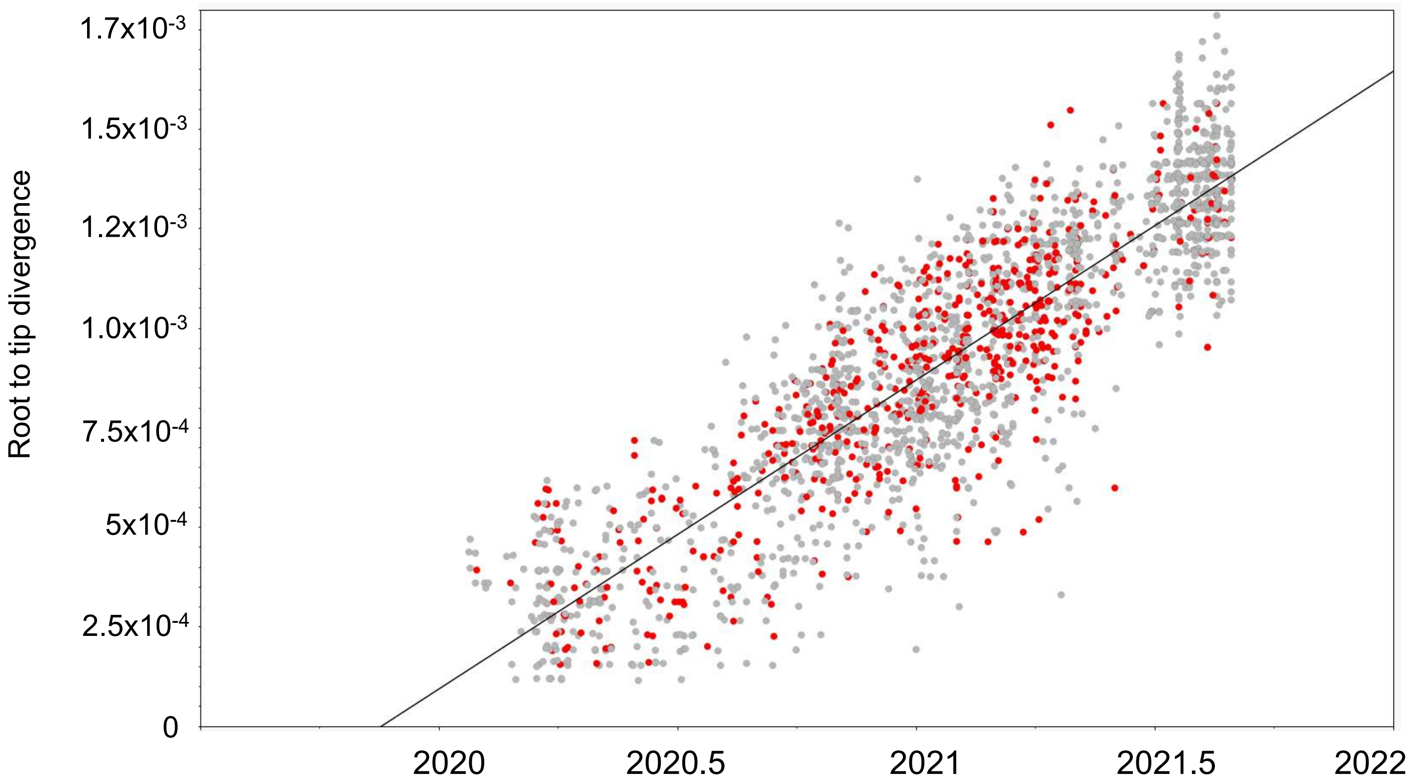

Supplementary Fig. 2. **Root-to-Tip Genetic Distance for SARS-CoV-2 Sequences in the Maximum Likelihood Tree Plotted against collection date.** The Pearson correlation coefficient between root-to-tip distance and collection date is 0.682. Sequences obtained by OPBG population aged ≤12 years are colored in red (OPBG population aged ≤ 12 years =red; GISAID and OPBG population aged >12 years = gray).

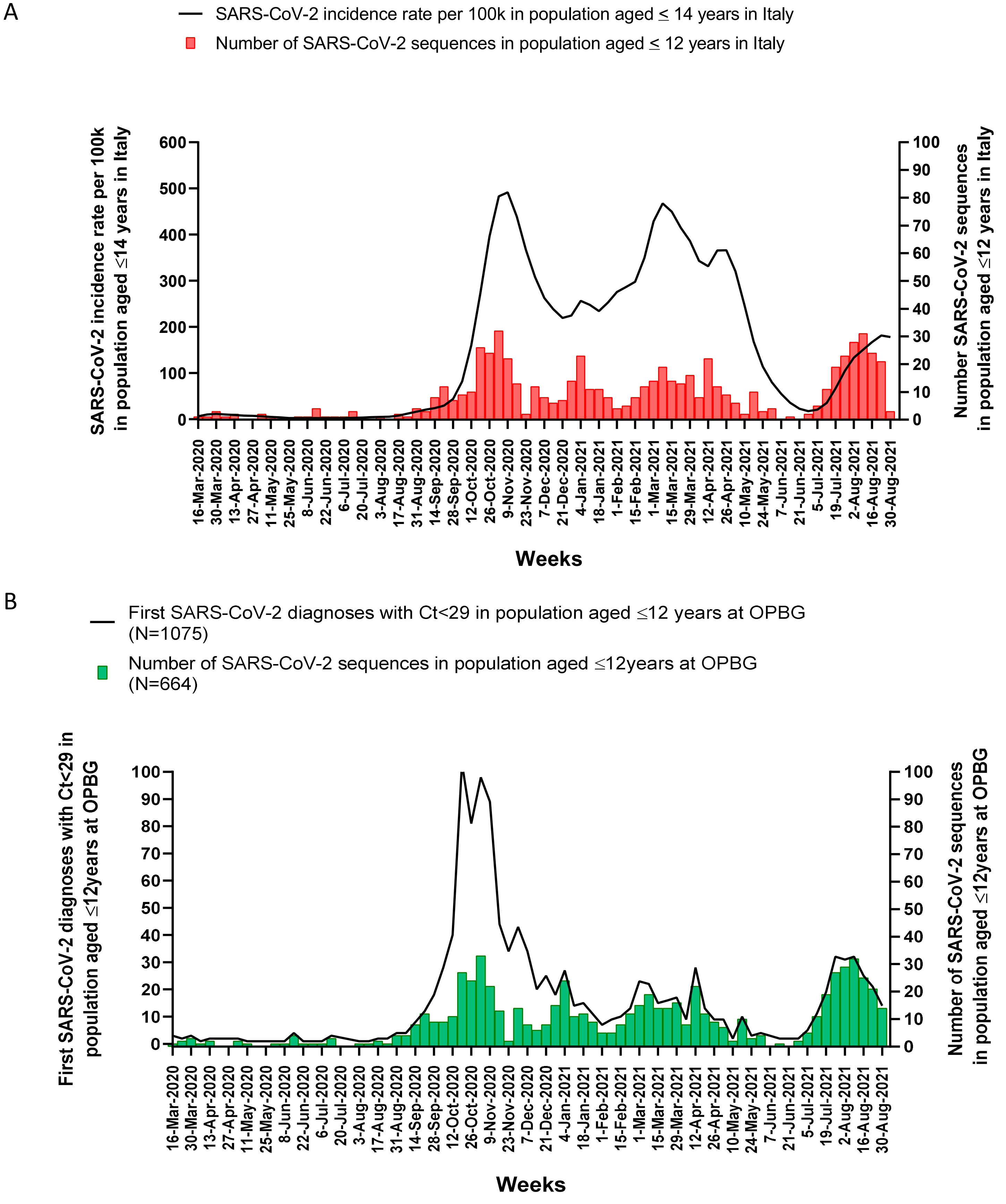

Supplementary Fig.3: **SARS-CoV-2 sequences obtained by population aged ≤12 in Italy against SARS-CoV-2 incidence rate in population aged ≤14 in Italy (A). SARS-CoV-2 sequences obtained by population aged ≤12 at OPBG against SARS-CoV-2 diagnoses with CT<29 in population aged ≤12 at OPBG (B).**

Information regarding incidence rate were made available on ECDC web page https://www.ecdc.europa.eu/en/publications-data/covid-19-data-14-day-age-notification-rate-new-cases
